# Fibroblast growth factor 23 contributes to diminished bone mineral density in childhood inflammatory bowel disease

**DOI:** 10.1186/1471-230X-12-44

**Published:** 2012-05-02

**Authors:** Mostafa Abdel-Aziz El-Hodhod, Ahmad Mohamed Hamdy, Amal Ahmed Abbas, Sherine George Moftah, Alhag Ahmed Mohamed Ramadan

**Affiliations:** 1 Department Pediatrics, Faculty of Medicine, Ain Shams University, Cairo, Egypt; 2 Department Clinical & Chemical Pathology, Faculty of Medicine, Ain Shams University, Cairo, Egypt; 3 Department Radiodiagnosis, Faculty of Medicine, Ain Shams University, Cairo, Egypt; 4 Pediatrician, Ministry of Health, Cairo, Egypt

**Keywords:** IBD, Children, FGF23, Bone mineral density

## Abstract

**Background:**

Diminished bone mineral density (BMD) is of significant concern in pediatric inflammatory bowel disease (IBD). Exact etiology is debatable. The recognition of fibroblast growth factor 23 (FGF23), a phosphaturic hormone related to tumor necrosis factor alpha (TNF-α) makes it plausible to hypothesize its possible relation to this pathology.

**Methods:**

In this follow up case control study, BMD as well as serum levels of FGF23, calcium, phosphorus, alkaline phosphatase, creatinine, parathyroid hormone, 25 hydroxy vitamin D_3_ and 1, 25 dihydroxy vitamin D_3_ were measured in 47 children with IBD during flare and reassessed in the next remission.

**Results:**

Low BMD was frequent during IBD flare (87.2%) with significant improvement after remission (44.7%). During disease flare, only 21.3% of patients had vitamin D deficiency, which was severe in 12.8%. During remission, all patients had normal vitamin D except for two patients with Crohn’s disease (CD) who remained vitamin D deficient. Mean value of serum FGF23 was significantly higher among patients with IBD during flare compared to controls. It showed significant improvement during remission but not to the control values. 1, 25 dihydroxy vitamin D_3_, FGF23, serum calcium and urinary phosphorus were significant determinants of BMD in IBD patients.

**Conclusions:**

We can conclude that diminished BMD in childhood IBD is a common multifactorial problem. Elevated FGF23 would be a novel addition to the list of factors affecting bone mineral density in this context. Further molecular studies are warranted to display the exact interplay of these factors.

## Background

Children with inflammatory bowel disease demonstrate decreased bone mineral density [1]. Risk factors include the use of steroids [2], defective nutritional status [3] and the inflammatory process itself [4]. While some studies recommend the use of oral vitamin D and calcium for prevention of bone loss in patients with IBD [5], no sufficient evidence exists of vitamin D deficiency in these patients [6-8] or of their benefit from vitamin D supplementation [9].

Fibroblast growth factor 23 (FGF23) belongs to the group of phosphatonins, which enhance renal phosphate excretion and inhibit renal 25-hydroxy-vitamin D3 1α hydroxylase [10]. Increased FGF 23 secretion was proved to be the mechanism of bone disease in many diseases including tumor induced osteomalacia [11] and X-linked hypophopshatemic rickets [12]. Decreased degradation of FGF23 was found to be the mechanism of bone disease in autosomal dominant hypophosphatemic rickets [13]. Impaired renal excretion of FGF23 was proved to be the mechanism of renal osteodystrophy and post-transplant hypophosphatemia [14].

The PHEX (phosphate-regulating with homologies to endopeptidases on the X chromosome) gene encodes a Zinc-metalloendopeptidase expressed primarily in osteoblasts and odontoblasts. Studies seemed to confirm the hypothesis of proteolytic degradation of FGF-23 by PHEX [15]. The down-regulation of the PHEX gene results in decreased degradation of FGF-23 and a subsequent increase in its circulating levels [16].

TNF-α is the most recognized cytokine in the pathogenesis of IBD [17]. Uno et al. demonstrated that in experimental animals, TNF-alpha decreases Phex mRNA and protein expression via a transcriptional mechanism and this is at least in part responsible for inhibition of osteoblast mineralization [18].

### Hypothesis

We hypothesize that FGF23 is increased in patients with IBD and, being a phosphaturic hormone, may contribute to the diminished BMD noticed in this patient population. So the aim was to assess relation of serum FGF-23 to BMD among children with IBD.

## Subjects and methods

This follow up case control clinical study included 47 children with IBD (27 with ulcerative colitis "UC" and 20 with Crohn’s disease "CD") recruited from amongst IBD patients followed up at the Pediatric Gastroenterology Unit, Ain Shams University Faculty of Medicine over the period from December 2008 to December 2010. They were 32 males (14 UC and 18 CD) and 15 females (13 UC and 2 CD), with a mean age of 11.6 ± 3.5 years. Patients were studied during disease activity, either at initial diagnosis (6 UC and 5 CD) or in a disease relapse (21 UC and 15 CD). Patients underwent clinical and laboratory reassessment 3 months after achieving remission depending on feasibility of follow up visits and availability of required tests as DXA scan. The duration between the initial evaluation "flare" and reassessment "remission" ranged between 4 and 9 months (mean of 7.12 +/− 2.8 months). Fifty healthy, age and sex matched children (12 males and 9 females with an age range of 4–16 years and a mean age of 12.8 ± 3.77 years) were included as a control group.

### Inclusion criteria

1- Diagnosis of IBD based on the Porto criteria [19].

2- Disease flare that was assessed using Pediatric Ulcerative Colitis Activity Index (PUCAI) for UC [20] and modified Pediatric Crohn’s disease Activity Index (PCDAI) for CD [21].

3- No steroid therapy for at least three months prior to enrollment in this study.

### Exclusion criteria

1- Critically ill patients who cannot be transferred for DXA procedure.

2- Concomitant endocrinal, renal or genetic bone diseases.

### Plan of work

After approval of the study by the local ethics committee, informed consents were taken from the guardians of all patients, as well as assents from older children.

A full medical history and general examination, including anthropometric assessment of height for age with its Z score as well as body mass index (BMI), were done [22].

Laboratory assessment included complete blood count [Coulter 1660], C-reactive protein [AVITEX- CRP latex test [23]], ESR [Westergren method [24]], serum calcium [colorimetric method [25]], serum alkaline phosphatase [autoanalyzer], serum phosphorus [direct UV method without reduction [26]] and urinary phosphorus [colorimetric methods using an autoanalyzer (Multistat III Plus, Instrumentation Laboratory, Inc, Lexington, MA)]. Colonoscopy and esophago-gastrodudenoscopy [Pentax videosopes, PENYAX GmbH CO. Juulius Vosseler Strasse 104, 22527 Hamburg, Germany] with histopathologic assessment of multiple biopsies were done for all patients. Serum creatinine was measured using Synchron cx7 (Beckman Inc.).

Measurement of 25 (OH) D_3_ was done using radioiodine (^125^I)-based RIA kits (DiaSorin, Stillwater, MN) [27] with unit of measurement being ng/ml. Values below 15 ng/ml were considered indicative of vitamin D deficiency [28] and values below 8 ng/ml were considered indicative of severe vitamin D deficiency [29]. Measurement of 1, 25 (OH)_2_ D_3_ was done using Human 1, 25-Dihydroxy-Vitamin D RIA Kit (IBL International GmbH) with unit of measurement being pg/ml [30]. PTH level was measured by the Immulite 2000 Intact PTH assay.

Serum FGF23 was measured using a C-terminal human FGF23 ELISA (Immunotopics, San Clemente, CA) [31]. The plasma was stored at –20 C till assessment at end of study. It was expressed as a concentration of pg/ml with a sensitivity of 4 pg/ml.

### Bone densitometry

BMD was determined by DXA Lunar scan [DPX-MD 2001, GE medical systems, USA]. Device was calibrated daily, and the technical error calculated to be less than 1%. Z-scores were calculated from BMD values using the reference data for bone density for age and sex [32] and corrected to bone age which was assessed from X Rays of the left hand [33]. The values of total body BMD were used for analysis.Values between −1.0 and −2.5 denoted a mild decrease in BMD and < −2.5 were diagnostic of severe decrease [34].

#### Treatment

For induction of remission**,** all patients received oral prednisone (1–2 mg/kg/day) for 3–4 weeks. Parenteral antibiotics and other supportive measures were individually adjusted. After induction of remission, patients were then maintained on 5 amino salicylic acid [35]. Six CD patients subsequently received infliximab as add-on therapy [36]. One patient with UC underwent total proctocolectomy with ileo-anal anastomosis [37]. Remission was defined as PUCAI < 10 points for UC [20] and PCDAI < 15 points for CD [21].

Nutritional support after enrollment in the flare state included calcium (500 – 1000 mg daily) and oral vitamin D_3_ supplementation as 1000 IU daily for non-deficient and 10000 IU daily for deficient children.

Regarding the physical activity, patients, prior to flare, were not bed ridden but were ambulant and most of them were attending their full-day school activities. The hospital admission ranged between 3–4 weeks. The remaining time till reassessment was spent with their normal range of activity with the exception of strenuous exercises. An exception was one patient with CD who had multiple vertebral fractures with marked decrease of BMD. However we did not quantify physical activities to consider them in analysis.

### Statistical methods

The computer program SPSS for Windows, release 10.0 (SPSS, inc, Chicago, IL, USA) was used for data entry and analysis. All quantitative data were analyzed as mean and standard deviation (SD). Student’s t test was used to compare quantitative data between different groups. Chi square test was used to compare qualitative variables between different groups. Multiple regression analysis was performed.

## Results

The duration of illness from definitive diagnosis of IBD ranged from 0 to 48 months, while the duration from the first complaint till inclusion in the study ranged from 6 to 60 months.

Patients of the UC group were significantly older than the CD group (12.77 +/−1.71 and 10.49 +/− 3.34 years respectively). Both were comparable to the control group (11.86 +/−3.39 years; p value 0.228 and 0.202 respectively). The male to female ratio was 18:2 in the CD group, and 14:13 in the UC group (p = 0.006).

Bleeding per rectum and joint involvement (arthralgia or arthritis) were more frequently encountered among patients with UC (100%, 48.15% and 48.15% respectively) than CD (35%, 10% and 15% respectively). One of the included patients had pathological fractures of the last 3 lumbar spines.

The mean values of Z score of height-for-age was significantly lower in children with disease activity compared to controls (p < 0.001). Both groups showed a non-significant improvement after remission which remain significantly lower than controls (p < 0.001).

BMI was significantly lower in UC and CD patients during flare (17.26 +/− 2.34 and 17.13 +/− 2.46 respectively) and remission (19.27 +/− 2.07 and 20.27 +/− 3.18 respectively) than in the control group (25.43 +/−2.65) (p < 0.001). The difference between flare and remission was significant for both UC (p = 0.002) and CD (p = 0.001).

Bone mineral density and Z score of corrected BMD to bone age and sex were both significantly lower during disease activity compared to values at remission (Table 1). Severe affection (Z score < −2.5) was present in 88.9% and 75% of patients with active UC and CD respectively. After remission, this decreased to 37% and 10% of patients with UC and CD, respectively (Table 2). The rate of change of Z score of corrected BMD to bone age showed no significant difference between the UC and CD groups (Table 3).

**Table 1:** Comparisons of biochemical laboratory bone markers among patients and controls

		**Control (I)**	**UC Flare (IIa)**	**UC Remission (IIb)**	**IIa vs IIb t (p)**	**CD Flare (IIIa)**	**CD Remission (IIIb)**	**IIIa vs IIIb t (p)**
BMD (g/cm^2^)			0.74 +/−0.14	0.91 +/−0.11	−5.15	0.73 +/−0.13	0.92 +/−0.12	−4.34
					(<0.0001)			(<0.0001)
Z score for BMD			−3.47 +/−1.75	−1.02 +/−1.21	−5.97	−3.24 +/−2.20	0.06 +/−1.48	−5.57
					(<0.0001)			(<0.0001)
25 (OH) VD_3_(ng/ml)		47.14+/−11.78	37.41 +/−16.69	40.96 +/−12.53	−0.89	34.40 +/−19.21	42.00 +/−15.49	−1.38
					(0.38)			(0.176)
	Comparison with control	T	2.27	−1.74		2.58	1.2	
		(p)	(0.028)	(0.089)		(0.014)	(0.237)	
1, 25 (OH)_2_ VD_3_ (pg/ml)		30.86 +/−6.67	56.11 +/−12.11	30.96 +/−8.14	8.96	65.65+/−14.99	22.80+/−3.70	12.403
					(<0.0001)			(<0.0001)
	Comparison with control	T	8.59	0.05		9.68	−4.75	
		(p)	(<0.0001)	(0.96)		(<0.0001)	(<0.0001)	
FGF23 (pg/ml)		16.90+/−4.91	62.22 +/−20.70	26.89 +/−8.83	8.157	67.75 +/−18.36	23.55 +/−9.95	9.465
					(<0.0001)			(<0.0001)
	Comparison with control	T	9.80	4.64		12.25	2.73	
		(p)	(<0.0001)	(<0.0001)		(<0.0001)	(0.009)	
PTH		38.410 +/−19.98	67.71 +/−20.930	55.32 +/−17.23	0.81	62.62 +/−23.07	51.81+/−20.58	0.65
					0.42			0.52
			−5.01	−3.21		−3.68	−2.17	
			0.0001	0.002		0.001	0.036	

**Table 2:** Frequency of osteopenia and osteoporosis in flare and remission of UC and CD patients

		**Normal BMD (BMD > −1)**	**Abnormal BMD**	**Mild degree (BMD −1 to −2.5)**	**Severe degree (< −2.5)**	**Chi squre (P)**
UC	Flare	3 (11.1%)	24 (88.9%)	0	24 (88.9%)	6.17
	remission	11 (40.7)	16 (59.3%)	6 (22.2%)	10 (37%)	(p = 0.013)
CD	Flare	3 (15%)	17 (85%)	2 (10%)	15 (75%)	14.55 (p < 0.0001)
	remission	15 (75%)	5 (25%)	3 (15%)	2 (10%)	
All IBD	Flare	6 (12.77%)	41 (87.2%)	2 (4.23%)	39 (82.98%)	18.95 (p < 0.0001)
	remission	26 (55.32%)	21 (44.7%)	9 (19.15%)	12 (25.53%)	

**Table 3:** Comparison of rate of changes of Z score of BMD between remission and flare among patients with UC and CD

**Parameter**	**Rate of change in UC Mean (as%) (+/− SD)**	**Rate of change in CD Mean (as%) (+/− SD)**	**t**	**p**
Z score of BMD corrected to bone age.	−85.43 +/− 60.94	−111.53 +/− 62.13	1.826	0.074

Serum calcium was significantly lower in UC and CD patients during both disease activity (9.45 +/− 0.45 and 9.26 +/− 0.34 mg/dl respectively) and after disease remission (9.55 +/− 0.43 and 9.500 +/− 0.67 mg/dl respectively) compared to the control group (9.78 +/− 0.26 mg/dl) (p < 0.001). Although mean serum calcium increased after remission, it was not statistically different from that during remission (p > 0.05 in both groups).

Serum phosphorus was significantly lower during disease activity in UC and CD patients (3.40 +/− 1.14 and 3.57 +/− 0.93 mg/dl respectively) compared to remission values (5.16 +/− 0.54 and 5.10 +/− 0.48 mg/dl respectively) and the control group (5.22 +/− 0.48 mg/dl) (p < 0.001). Values during remission were not different from those of controls (p > 0.05).

Serum alkaline phosphatase was significantly higher in UC and CD patients during flare compared to values during remission and controls with p values of <0.0001 for all. However, remission values of both groups were not different from control group.

Similarly, urinary phosphorus was significantly higher during flare in both UC and CD patients (2.01 +/− 0.77 and 2.06+/− 0.69 g/24 hours urinary output respectively) compared to remission (0.66 +/− 0.26 and 0.61+/− 0.18 g/24 hours urinary output respectively) and controls (0.74 +/− 0.19 g/24 hours urinary output) (p < 0.001). Values during disease remission were not different from control ones.

Serum creatinine was not different between cases (flare or remission) and controls.

The 25 hydroxy vitamin D_3_ values were significantly lower during disease flare compared to control values. Remission values were not significantly different from values during disease activity, or from control values (Table 1). On the other hand, 1, 25 (OH)_2_ vitamin D_3_ values were significantly higher during flare of UC and CD compared to controls (Table 1).

FGF23 serum levels were significantly higher during disease flare in UC and CD patients compared to controls. Although values significantly decreased during remission, they remained higher than controls (Table 1).

Serum parathyroid hormone was significantly higher in UC flare and remission as well as CD flare and remission than controls. Flare of UC showed significant higher values than their remission values (p = 0.019). Flare and remission values in CD were not different. Comparison of UC versus CD showed no significant differences.

Regression analysis in the ulcerative colitis group during flare showed the only significant determining factors were FGF23 followed by serum calcium. Regression analysis of BMD in CD group during flare (adjusted R2 = 0.971) showed many significant determining factors affecting BMD. On top comes 1, 21 (OH)2 VD, followed by urinary phosphorus and FGF23 (P <0.0001 for all).

Although the number of CD patients treated with infliximab is very small; the analysis of their BMD and FGF23 showed interesting results. Z score for BMD showed dramatic improvement to normal values in the 6 patients (0.89 +/− 0.67) compared to their flare (−3.32 +/− 2.31) with p value <0.0001. They showed simultaneous decrease of FGF23 to completely normal values in remission (15.38 +/− (3.11)) compared to flare (69.23 +/− (19.34)) with a p value <0.0001.

## Discussion

### BMD

The diminished BMD during flare with significant improvement in remission agrees with previous similar results in IBD patients using either total body or lumbar spine bone mineral density [38-40]. Herzog et al. [41] and Ahmed et al. [42] reported significant values of 26% and 22% respectively. Schmidt et al. [40] showed no difference between UC and CD regarding the frequency of low BMD.

This degree of improvement was achieved in a period of 7.12 +/− 2.8 months between the flare and remission assessment. Improvement of BMD in such a relatively short period is consistent with Viapiana et al. [43] who found that a 3 months period of intensive nutritional therapy in anorexia nervosa was sufficient to show the significant improvement in BMD. Similarly, Bhambri et al. [44] found that BMD measured by DXA was significantly improved but not to base line after therapy for 2.8+/−1.4 months.

The residual diminished BMD in a significant proportion of IBD children, during remission, suggests that nutritional therapy, IBD specific therapy and/or their duration are not sufficient to restore BMD to complete normality.

### Vitamin D

The mean serum level of 25 (OH) vitamin D_3_, as a measure of vitamin D status [45], showed significantly lower values during flare of UC and CD compared to controls. Deficiency (< 15 ng/ml) or severe deficiency (< 8 ng/ml) were uncommon among patients with IBD during flare and markedly improved after remission. The vitamin D nutritional state returned to normal after achieving disease remission, although the intake of vitamin D and calcium had not changed. This may indicate that the state of hypovitaminosis D is a reflection of disordered bioavailability with disease activity rather than isolated nutritional deficiency. The normalization of vitamin D nutritional status with residual low bone mineral density in IBD patients implies that VD is not the sole player in the development of IBD osteopathy. Sentongo et al. [28] found that there was no association between hypovitaminosis D and bone mineral density (P = 0.10) and Hessov et al. [6] found reduction in trabecular bone mass in the presence of normal mean serum levels of the three vitamin D metabolites (25-hydroxyvitamin D_3_, 24,25-dihydroxyvitamin D_3_, and 1,25-dihydroxyvitamin D_3_) after ileal resection in CD. Higher values of 1,25- dihydroxyvitamin D_3_ during flare of UC and CD may be due to inflammation-induced intestinal over-expression of activating enzymes. Abreu et al. [8] also demonstrated high levels of 1,25- dihydroxyvitamin D_3_ in IBD patients, more so in CD patients. They explained this by demonstrating an increased expression of intestinal 1 alpha hydroxylase in CD patients which would contribute to increased activation of vitamin D. This may be reinforced by our finding that levels of active form were dramatically reduced to control values or even less after achieving disease remission.

### FGF23

FGF23 was significantly higher during disease flare in both UC and CD groups, and while significantly decreasing during remission, did not return back to normal control values. Although phosphatonins including FGF23 inhibit 1 α hydroxylase enzyme in the kidney [10] yet, the high levels of active vitamin D in this study minimizes this role in our context

This finding was not reported in previous clinical studies on IBD patients. It would be related to the high values of TNF alpha reported in such patients [17]. Uno et al. [18] demonstrated that in experimental animals, TNF-alpha decreases Phex mRNA and protein expression via a transcriptional mechanism and this is at least in part responsible for inhibition of osteoblast mineralization. The Phex mRNA and protein is known to be involved in degradation of FGF23 [46]. So we can propose that higher values of FGF23 with its phosphaturic action [10] and the resultant hypophosphatemia may share, among other factors, in the process of diminished BMD in these particular patients. The high levels of FGF23 cannot be attributed to impaired renal functions as serum creatinine was within normal for all patients. 1,25-dihydroxyvitamin D_3_ may exert its largest effect on FGF23 expression/production when exposed to high levels of extracellular Pi in osteoblasts/osteocytes [47].

### PTH

Parathyroid hormone was found to be significantly higher during disease flare than in remission. Moreover, remission values are still significantly higher than in controls. The relation of PTH to bone metabolism is established. This establishes the role of altered PTH in the cascade of osteopathy seen in these cases. The FGF23 polypeptide also suppresses the expression of vitamin D 1-hydroxylase and PTH, resulting in a reduction in serum 1,25-dihydroxyvitamin D_3_[48] and PTH [49] levels. So our results, showing high 1, 25(OH)_2_ VD_3_ and PTH, suggest that FGF23 elevation is a secondary event rather than a primary incident. The TNF alpha role may augment this elevation through decrease of FGF23 degradation.

### The predictors of decreased BMD

The etiology of IBD related bone disease seems to be multi-factorial with inflammation itself being the most important factor [4]. In our present work, bone mineral density was low during disease flare despite absence of steroid therapy for at least 3 months prior to enrolment. It dramatically increased after remission even with the use of steroids in all patients. This may suggest a lesser role of steroids on the bone mineral density in IBD patients. Walther et al. [50] found a similar proportion of osteoporosis in steroid-naive (12%) and steroid-treated (11%) pediatric IBD patients. Paganelli et al. [4] reported that the correlation between the lifetime cumulative dose of steroids or duration of treatment and BMD was not statistically significant.

Regression analysis for different clinical and laboratory variables in the ulcerative colitis showed that the only significant determining factors were FGF23 followed by serum calcium. On the other hand in CD, it showed many significant determining factors affecting BMD. On top comes 1,25-dihydroxyvitamin D_3_, followed by urinary phosphorus and FGF23. The significant role of vitamin D in CD rather than in UC group may reflect a problem related to inflammation or absorption in the former group.

Previous studies have found that BMI correlated with BMD in IBD both in children [39]. But our analysis did not show an impact of BMI on the BMD in view of other variables.

The marked improvement noted in our CD group might be related to use of infliximab, a TNF inhibitor, in some of them. Franchimont et al. [51] and Ryan et al. [52] found that Infliximab rapidly improves bone formation in adults with IBD.

Controversy exists about the relation between decreased BMD and fracture risk among children with IBD [53]. However, low BMD during childhood is associated with increased fracture risk later in life [54]. Bone metabolism in children is characterized by predominant bone modeling with simultaneous activity of both osteoblasts and osteoclasts on different parts of the bone. Therefore, the principles of bone loss observed in adult patients with chronic inflammation are not directly applicable to children [55]. Understanding the specific underlying mechanisms for decreased BMD among children with IBD may help to implement more effective therapies for this complication.

## Conclusion

We can conclude that BMD is significantly low in IBD during disease flare with significant improvement after remission. This osteopathy is multifactorial with a proposed new place for the elevated FGF23 levels in such a complex interplay.

## Abbreviations

IBD, Inflammatory bowel disease; BMD, Bone mineral density; FGF23, Fibroblast growth factor 23; CD, Crohn’s disease; TNF-α, Tumor necrosis factor alpha; PHEX, Phosphate-regulating with homologies to endopeptidases on the X chromosome; UC, Ulcerative colitis; PUCAI, Pediatric ulcerative colitis activity index; PCDAI, Pediatric Crohn’s disease activity index; BMI, Body mass index.

## Competing interests

The authors declare that they have no competing interests.

## Authors’ contributions

MAE idea of the work, some endoscopies, discussion of results and revision of the manuscript. AMH some endoscopies, recruitment of patients, clinical assessment and therapy, statistical analysis and writing the manuscript. AAA the laboratory workup. SGM the DXA scan. AAMR literature review and assisted in recruitment of patients. All authors read and approved the final manuscript.

## Pre-publication history

The pre-publication history for this paper can be accessed here:

http://www.biomedcentral.com/1471-230X/12/44/prepub
